# Multisite clinical evaluation of the iFAST rapid antimicrobial susceptibility test direct from positive blood cultures containing gram-negative organisms

**DOI:** 10.1128/jcm.00558-26

**Published:** 2026-08-31

**Authors:** Michael Hornsey, Isobel Taylor, Toby King, Zoe Brown, Daniel Nathan Tomás Cançado, Katie Judd, Eleanor Kuszynski, Rowena Lines, Rhiannon Lyon, Xiang Wang, Allyson Lloyd, Robert Impey, Gareth Ashworth, Maria Gatenby, Alasdair MacGowan, Mahableshwar Albur, Alan Noel, Marie Attwood, Pippa Griffin, Cliodna Brennan, Freddie Oubridge Brock, Jane Stewart, Estíbaliz Alonso-Moreno, Hywel Morgan, Daniel Spencer

**Affiliations:** 1 iFAST Diagnostics, Solent Business Park, Fareham, United Kingdom; 2 Queen Alexandra Hospital, Portsmouth Hospitals University NHS Trust 6698 , Portsmouth, United Kingdom; 3 pKpD Laboratory, Severn Infection Sciences, Southmead Hospital North Bristol NHS Trust, Bristol, United Kingdom; 4 Southampton General Hospital, University Hospitals Southampton 105634, Southampton, United Kingdom; 5 School of Electronics and Computer Science, Institute for Life Science, University of Southampton 152287, Southampton, United Kingdom; Mayo Clinic, Rochester, Minnesota, USA

**Keywords:** rapid diagnostic, antimicrobial susceptibility test, antimicrobial resistance

## Abstract

iFAST is a label-free rapid antimicrobial susceptibility test (AST) that delivers qualitative results in under 4 h from a positive blood culture bottle. The technique measures changes in the electrical properties of thousands of individual bacteria following exposure to antibiotics. We report the diagnostic accuracy from the first clinical study of the iFASTone rapid AST system in three different centers according to ISO 20776-2:2021. Compared to the reference laboratory broth microdilution method, categorical agreement was 97.8% and 97.2% for the *Enterobacterales* and non-fermenting gram-negative species, respectively. iFAST specificity and sensitivity for the *Enterobacterales* were 98.2% and 96.8%, respectively, and 96.9% and 98.4% for the non-fermenting species, exceeding the acceptance criteria (≥95% sensitivity and specificity). Reproducibility was assessed with 12 isolates across 3 sites, in triplicate, on 3 separate days, with different operators at each site. Agreement was 95.7%.

**IMPORTANCE:**

Even short delays in the initiation of antimicrobial therapy in patients with bloodstream infections are associated with increased morbidity and mortality. Consequently, current guidelines recommend initiation of empiric antimicrobial therapy within 1 h of presentation. However, empiric broad-spectrum therapy may not always be appropriate and can contribute to the selection of resistant organisms. Antimicrobial susceptibility testing (AST) is therefore critical for antimicrobial stewardship and the timely selection of targeted therapy, but conventional methods typically require 1 to 2 days to generate results. This study evaluated the diagnostic accuracy of iFAST, a new low-cost, label-free rapid AST using 375 *Enterobacterales* and non-fermenter isolates tested directly from positive blood cultures, according to ISO-20776-2:2021. Compared with broth microdilution, iFAST demonstrated high sensitivity, specificity, and categorical agreement, with a time to result of less than 4 h. These findings support the implementation of iFAST for same-shift AST results.

## INTRODUCTION

Bloodstream infections (BSIs) and sepsis continue to be a leading cause of morbidity and mortality worldwide, accounting for millions of deaths each year (1). The global burden of sepsis is estimated to be nearly 49 million cases annually, with 11 million deaths, accounting for nearly 20% of global deaths, with approximately 85% of all sepsis-related deaths in low- and middle-income countries (1, 2). Surviving Sepsis International Guidelines recommend initiation of empiric antibiotic therapy within 1 h of presentation to hospital (3). Even an hourly delay can lead to increased mortality, prolonged hospitalization, and higher healthcare costs (4). Inappropriate or delayed therapy is linked to worse clinical outcomes, longer hospital stays, and escalating healthcare expenditure. However, the widespread and inappropriate use of empiric broad-spectrum antibiotics contributes to the selection and spread of antimicrobial resistance.

Blood culture remains the key investigation for sepsis and a gold standard for diagnosing bloodstream infections (5). However, conventional antimicrobial susceptibility testing (AST), including broth microdilution (BMD) and standard disk diffusion methods, typically requires 24–48 h after blood culture positivity to yield results. Traditional approaches are generally too slow to impact early therapeutic decision-making. Therefore, several novel rapid diagnostic technologies providing a shorter time to result (TTR) have been developed and are the subject of recent reviews (6, 7). Rapid pathogen identification directly from positive blood cultures (PBCs) is frequently performed with matrix-assisted laser desorption ionization-time of flight mass spectrometry (MALDI-TOF MS) and/or molecular diagnostic systems. Some of these assays detect resistance-associated genes, but phenotypic response to antibiotics may not be fully predicted by genotypic data. The absence of a resistance gene does not necessarily confirm phenotypic susceptibility, as gene expression and regulatory factors can influence antimicrobial response. Therefore, rapid phenotypic AST, which directly measures bacterial behavior following antibiotic exposure, remains an important component of the diagnostic pathway, particularly in light of evidence that rapid identification alone does not improve patient outcomes (8).

For such rapid AST systems to achieve broad clinical adoption, they must demonstrate analytical accuracy comparable to reference methods, support an expanding panel of clinically relevant antimicrobials, and integrate seamlessly into routine laboratory workflows. Additional considerations include cost-effectiveness, scalability, minimal hands-on time, inexpensive consumables with room temperature storage, and long shelf life, along with suitability for diverse healthcare settings, including resource-limited environments where access to diagnostic infrastructure remains a major barrier to optimal sepsis care.

Given the increasing global burden of antimicrobial resistance and the critical importance of early effective therapy in BSIs, there is a pressing need for rapid, reliable, and cost-effective phenotypic susceptibility testing directly from PBCs.

Several rapid AST systems have recently been introduced. The Accelerate Pheno system (9, 10) performs both identification and AST directly from PBCs within 7 h. It uses fluorescence *in situ* hybridization for identification and automated optical imaging to determine bacterial growth rates. Other optical analysis systems include the Gradientech QuickMIC, which uses an antibiotic gradient on a solid medium in a microflow cell to deliver a minimal inhibitory concentration (MIC) in 4 h (11, 12). The Q-linea ASTar system is an automated phenotypic test where growth is continuously monitored, giving an MIC 6 h after incubation (13, 14), with a similar TTR to the Quantamatrix dRAST (15, 16). The VITEK REVEAL uses novel sensors to measure volatile organic compounds, detecting bacterial growth by metabolomic profiling. It determines an MIC from a PBC in real time, taking between 3 and 8 h (17, 18). FASTinov has developed a rapid (2 h) phenotypic test that uses fluorescent viability dyes measured with flow cytometry to determine an AST result after a very short 1 h incubation time (19–21). The Selux DX Next-Generation Phenotyping AST system includes a sample preparation device that automatically prepares a McFarland standard from a PBC. It also uses fluorescent dyes to measure the metabolic viability and morphological surface area of bacteria. Blood processing takes 1 h for up to two specimens, and results are available in 5–6 h after incubation (22). Finally, the European Committee on Antimicrobial Susceptibility Testing (EUCAST) has developed an inexpensive, rapid, disk diffusion-based protocol (rapid antimicrobial susceptibility testing [RAST]) that can provide categorical susceptibility results directly from PBCs within 4–8 h, although fewer than 90% of inhibition zones are readable after 4 h of incubation (23).

iFAST is a rapid and low-cost phenotypic AST system that can provide clinically actionable results in just a few hours from isolates or a positive blood bottle. The method is based on measuring the electrical properties of thousands of single bacteria after a 2 h exposure to a panel of different antibiotics (24, 25). The measurement time is short (30 seconds), and the method mirrors BMD and does not require expensive reagents or complex sample preparation.

The iFASTone system developed by iFAST Diagnostics Ltd (United Kingdom) is an automated system for determining qualitative AST results for the most clinically significant gram-negative aerobic and facultatively anaerobic microorganisms direct from PBCs providing same-day results. Antimicrobial susceptibility or resistance is determined by comparing antibiotic-exposed samples with an unexposed control group. The hardware consists of a robotic fluid handling system, a microfluidic reading chip, electronics to create and amplify signals, software to analyze the signals, together with the reagents and consumables required to perform the test. Custom algorithms are used to determine the susceptibility of an organism to a range of different antimicrobials as in other commercial systems.

Here, we describe the first multisite clinical evaluation of the iFASTone system for gram-negative AST performed directly from PBCs, using reference BMD as the comparator. The study was designed and conducted in accordance with the priniciples of ISO-20776-2:2021 (*Clinical laboratory testing and in vitro diagnostic test systems - Susceptibility testing of infectious agents and evaluation of performance of antimicrobial susceptibility test devices*).

## MATERIALS AND METHODS

The evaluation was performed on the iFAST gram-negative panel, which consisted of 15 antimicrobials commonly used to treat bloodstream infections (BSIs) (Table 1). The antimicrobial-organism combinations encompass both *Enterobacterales* and non-fermenting gram-negative bacteria, providing coverage of a broad range of clinically significant pathogens (Table 1). The panel is a standard 96-well plate format and contains vacuum-dried antibiotics, stored at ambient temperature with a shelf-life of 2 years. The panel includes both commonly used first-line agents and reserve antibiotics suitable for empirical, escalation, or de-escalation antimicrobial therapy in the United Kingdom and elsewhere.

**TABLE 1:** List of antimicrobial-organism combinations indicated on the iFASTone system

Antimicrobial agents	Class	Abbreviation	*Escherichia coli*	*Klebsiella pneumoniae*	*Klebsiella oxytoca*	*Enterobacter cloacae* complex	*Serratia marcescens*	*Pseudomonas aeruginosa*	*Acinetobacter baumannii*
Ampicillin	Penicillin	AMP	X^*a*^						
Amoxicillin-clavulanic acid	Penicillin + clavam inhibitor	AMC	X	X	X				
Piperacillin-tazobactam	Penicillin + penicillanic acid sulfone inhibitor	PTZ	X	X	X	X	X	X	
Aztreonam	Monobactam	AZT	X	X	X	X	X	X	
Cefotaxime	3rd-generation cephalosporin	CTX	X	X	X	X	X		
Ceftazidime	3rd-generation cephalosporin	CAZ	X	X	X	X	X	X	
Ceftazidime-avibactam	Cephalosporin + diazabicyclooctane inhibitor	CAA	X	X	X	X	X	X	
Ceftolozane-tazobactam	Cephalosporin + penicillanic acid sulfone inhibitor	CTA	X	X	X	X	X	X	
Meropenem	Carbapenem	MER	X	X	X	X	X	X	X
Imipenem	Carbapenem	IMP	X	X	X	X	X		X
Ertapenem	Carbapenem	ERT	X	X	X	X	X		
Amikacin	Aminoglycoside	AMK	X	X	X	X	X	X	
Gentamicin	Aminoglycoside	GEN	X	X	X	X	X		X
Ciprofloxacin	Fluoroquinolone	CIP	X	X	X	X	X	X	X
Trimethoprim-sulfamethoxazole	Folate pathway inhibitor	SXT	X	X	X	X	X		X

^*a*^ X, denotes an indicated antimicrobial-organism combination.

The reference BMD method was performed in line with ISO 20776-1:2019 using custom Sensititre plates (Thermo Fisher Scientific, East Grinstead, West Sussex, UK) at Southmead Hospital Bristol with isolates measured as technical triplicates. If no modal MIC was obtained, then the median result was used to determine a consensus MIC for each antimicrobial-organism combination. The consensus MIC results were interpreted using EUCAST criteria (breakpoint table v. 15, 2025) and compared with the iFAST AST result (26). In instances where triplicate BMD testing gave three different MIC values and the median value fell outside ± 1 dilution with the other two values (Essential Agreement, EA), then retesting in triplicate was performed. If repeat testing similarly yielded three different MIC values where the median value fell outside of EA with the other two values, then that result was excluded from all analyses on the basis that a consensus reference BMD result could not be established.

Sensitivity, specificity, and categorical agreement (CA) were determined and referenced to BMD. Categorical agreement was defined as the number of results where iFAST agreed with the reference method divided by the total number of results. Sensitivity was defined as the number of resistant results where both methods agree divided by the total number of resistant results by BMD. Specificity was defined as the number of susceptible results where both methods agree divided by the total number of susceptible results by BMD. For antimicrobial–organism combinations for which EUCAST set the susceptible (S) breakpoint at ≤0.001 mg/L, susceptible, increased exposure (I) results were used to calculate specificity and CA. Results were reported separately for *Enterobacterales* and non-fermenters (*Pseudomonas aeruginosa* and *Acinetobacter baumannii*) as per ISO 20766-2:2021.

Discrepancy resolution testing (DRT) was performed in accordance with the principles described in ISO 20776-2:2021. In the first steps, any discrepant result for a given antimicrobial-organism combination was repeated in triplicate on iFASTone. If discrepancies remained, the BMD was repeated in triplicate.

A total of 375 isolates were tested across three United Kingdom sites (Queen Alexandra Hospital Portsmouth [POR], Southmead Hospital Bristol [BRI] and iFAST, Whiteley, Hampshire [FAS]). Across the three sites, 101 contemporary, chronologically sampled gram-negative isolates (as defined in ISO 20776-2:2021) from routine clinical blood culture samples and 274 stock isolates were processed. Contemporary isolates tested at the iFAST site were provided by Southampton General Hospital, University Hospitals Southampton NHS Foundation Trust. Stock isolates were obtained from Southmead Hospital Bristol, Southampton General Hospital, and International Health Management Associates (USA).

Only one gram-negative isolate per patient was included for this study. Isolates from stored collections (stock isolates) were used to supplement the contemporary isolates with known or rare resistance mechanisms or species for which the antimicrobial agent is indicated but are not commonly isolated from PBCs in the United Kingdom. The number of isolates of each species tested at the three sites is detailed in Table 2.

**TABLE 2:** Summary of species tested at each of the three sites^*a*^

Organism	Contemporary isolates per site			Stock isolates per site			Total
	POR	FAS	BRI	POR	FAS	BRI	
*Escherichia coli*	15	30	18	21	20	16	120
*Klebsiella pneumoniae*	5	7	9	24	23	15	83
*Klebsiella oxytoca*	0	1	1	9	8	6	25
*Enterobacter cloacae* complex	5	1	2	11	12	8	39
*Serratia marcescens*	0	0	0	12	11	7	30
*Pseudomonas aeruginosa*	4	3	0	12	13	10	42
*Acinetobacter baumannii*	0	0	0	11	16	9	36

^*a*^ POR, Queen Alexandra Hospital, Portsmouth; FAS, iFAST Diagnostics; BRI, Southmead Hospital, Bristol.

All isolates used in this work prior to measurement were identified to species level using MALDI-TOF-MS by the clinical site. An organism ID is required for the instrument to generate susceptibility results.

All tests were performed using contrived blood cultures. Approximately 25 CFU of all contemporary and stock isolates were spiked into 10 mL of healthy human donor blood (Cambridge Bioscience, Cambridge, UK) containing 0.06% wt/vol sodium polyanetholesulfonate (Biosynth, Compton, Berkshire, UK), transferred into either BACTEC Plus (Becton Dickinson, Franklin Lakes, NJ, USA) or BacT/ALERT (bioMérieux, Marcy-l’Étoile, France) aerobic blood bottles, incubated overnight at 37°C, and then processed at 8:00 a.m. the following morning. Bacteria were extracted from blood culture bottles using the MBT Sepsityper IVD Kit (Bruker Daltonics GmbH, Bremen, Germany) according to the manufacturer’s instructions with the following additional step: after discarding the supernatant following the final centrifugation step, the bacterial cell pellet was resuspended in 2 mL of sterile saline in a clean tube.

The iFASTone system automatically processed the extracted bacteria to generate a standardized inoculum (5 × 10^5^ CFU/mL). The system counts the number of organisms in a given volume to determine CFU/mL. It also uses calibration beads (latex microparticles) as internal quality control (QC). The inoculum along with Mueller Hinton Broth was aliquoted into individual wells of the iFAST gram-negative antibiotic plate containing the dried antibiotics. The inoculated plate was then incubated externally in a standalone incubator for either 2 (*Enterobacterales*) or 3 h (*P. aeruginosa*) at 35°C. After incubation, the inoculated plate was returned to the instrument for analysis. Antimicrobial susceptibility or resistance was determined by comparing antibiotic-exposed samples with an unexposed control. Relevant EUCAST interpretive criteria were applied.

All isolates tested were stored at the testing laboratories. One specimen of each isolate was sent to the reference laboratory for testing by BMD according to ISO 20776-1:2019.

Since the study was non-interventional and iFAST results were not used for patient management, no patient information was collected, and an informed consent waiver was requested and approved.

Appropriate QC strains were tested on the iFASTone instrument each day that clinical isolates were tested. Depending on the identity of the clinical isolates, either ATCC 25922 and/or ATCC 27853 were employed. For BMD testing at the reference laboratory, the following QC strains were tested on each day that clinical isolates were: ATCC 25922, ATCC 27853, ATCC 35218, ATCC 700603, and NCTC 13443.

Scatter plot data were analyzed using proprietary algorithms that automatically compare several electrical parameters to determine resistance/susceptibility for a particular antimicrobial (25). Algorithms were optimized for each organism-antimicrobial combination.

Both iFASTone and standard of care (disk diffusion) TTR was recorded for all contemporary isolates provided by Southampton General Hospital. Disk diffusion was performed at the hospital (where blood cultures were processed during core hours, 7 days per week), while iFASTone testing was performed at the iFAST site. TTR information was available for 41 of 42 isolates. The contemporary isolate set comprised both *Enterobacterales* and non-fermenters. TTR for iFASTone was defined as the beginning of the Sepsityper extraction process to the point at which the results were reported by the instrument. TTR for disk diffusion was the time from reporting the Gram stain to the time the results were entered into the laboratory information management system.

## RESULTS AND DISCUSSION

A total of 32 isolates were excluded from the study for one of the following reasons: the organism failed to grow (*n* = 7); the isolate was identified as a duplicate (i.e., the isolate was already enrolled under a different identifier [*n* = 5]); the iFASTone control cell count was too low (*n* = 14); an incorrect species-level identification had been provided (*n* = 4); there was evidence of a mixed inoculum from purity plates (*n* = 2). Nine results from a total of 4,715 were removed from the analysis either because of classification instability (e.g., major error [ME] to minor error [mE]), or because no consensus BMD or iFASTone result could be established (Table 3).

**TABLE 3:** Results excluded from analysis

Sample ID^*a*^	Results removed	Reason
POR_0003	AZT	mE to VME
BRI_1008	PTZ	No BMD consensus
BRI_1019	IMP	mE to VME
BRI_1027	IMP	No iFAST modal result
BRI_1033	MER	mE to ME
BRI_1045	CTX	No BMD consensus
BRI_1053	SXT	mE to VME
BRI_1075	PTZ, CTA	No BMD consensus

^*a*^ The first 3 letters of the sample IDs correspond to the site code: POR, Queen Alexandra Hospital, Portsmouth; BRI, Southmead Hospital, Bristol.

Across all 4,715 AST results available for analysis, there were 31 very major errors (VMEs) and 17 MEs. For 18 VMEs and 6 MEs, the consensus BMD MIC was within ±1 dilution of the clinical breakpoint (Table 4). The overall VME, ME, and mE rates were 2.3%, 0.5%, and 2.1%, respectively (Table 4). A few isolates presented with more than one error, but there was a complex resistance mechanism background in all cases. For example, BRI_1067, a stock isolate of *Serratia marcescens*, had a VME for piperacillin-tazobactam and ertapenem, was both an ESBL (SHV-5) and carbapenemase (IMP-8) producer. BRI_1051, an *Enterobacter cloacae* stock isolate, co-produced NDM-1, OXA-1, and a novel ACT-type AmpC enzyme. This had VMEs for ertapenem and gentamicin. Carbapenemase-producing *Enterobacterales* remain uncommon in the United Kingdom, with national surveys estimating a prevalence of approximately 0.02% among *Enterobacterales* isolates (27). Surveillance data indicate that only a minority of carbapenemase-producing organism detections arise from sterile site infections such as BSIs (28). The agents with the highest number of VMEs were amoxicillin-clavulanic acid and ceftolozane-tazobactam. For amoxicillin-clavulanic acid, 60% of VMEs were within ±1 dilution of the breakpoint, and 90% were within ±2 dilutions. A similar pattern was previously reported in a performance evaluation study of the FASTinov AST system, which is based on the use of viability dyes and optical flow cytometry (20). Of note, only a single breakpoint concentration well was measured for amoxicillin-clavulanic acid during this study. It is anticipated that measuring additional wells that flank the breakpoint concentration will significantly improve performance. In a separate study evaluating the performance of the FASTinov FASTgramneg kit, a relatively high number of errors were reported for ceftolozane-tazobactam (19), which is consistent with the five VMEs observed here. Of these five VMEs, two were within ±1 dilution of the breakpoint, and four were within ±2 dilutions. The remaining VME was a stock isolate of *P. aeruginosa*, BRI_1037, which was shown to produce IMP-18, VIM-2, and OXA-395, in addition to the extended-spectrum chromosomal AmpC enzyme, PDC-3. The most recent surveillance data from England indicate that, compared with previous years, antimicrobial resistance among *Pseudomonas* spp. bacteremia isolates remained mostly stable in 2024, with resistance rates below 8% across major antibiotic classes (29). Of the 17 MEs, 8 were of piperacillin-tazobactam, 3 of which were within ±1 dilution of the breakpoint. FASTinov similarly reported a higher number of MEs for piperacillin-tazobactam compared with other agents (19).

**TABLE 4:** Summary of categorical results and errors listed by antimicrobial^*a*^

Antimicrobial agent	Specificity (n/N)		Sensitivity (n/N)		Categorical agreement		VMEs (VME>1)	VME rate (aVME rate)	MEs (ME>1)	ME rate (aME rate)	mEs	mE rate
	Enterobacterales	Non-fermenters	Enterobacterales	Non-fermenters	Enterobacterales	Non-fermenters						
AMP	100% (41/41)	na	97.5% (77/79)	na	98.3%	na	2 (1)/79	2.5% (1.3%)	None	na	na	na
AMC	98.1% (104/106)	na	91.8% (112/122)	na	94.7%	na	10 (4)/122	8.2% (3.3%)	2 (1)/106	1.9% (0.9%)	na	na
AZT	88.7% (165/186)	94.6% (35/37)	100% (104/104)	100% (5/5)	92.8%	95.2%	None	na	2 (1)/223	0.9% (0.4%)	24/337	7.1%
PTZ	95.6% (175/183)	94.9% (37/39)	98.2% (110/112)	100% (3/3)	96.6%	95.2%	2 (1)/115	1.7% (0.9%)	8 (5)/222	3.6% (2.3%)	2/337	0.6%
CTX	99.4% (180/181)	na	100% (107/107)	na	99.7%	na	None	na	None	na	8/296	2.7%
CAZ	92.3% (169/183)	86.8% (33/38)	99.0% (100/101)	100% (4/4)	94.7%	88.1%	None	na	1 (1)/221	0.5% (0.5%)	25/339	7.4%
CAA	100% (266/266)	100% (39/39)	100% (31/31)	66.7% (2/3)	100%	97.6%	1 (0)/34	2.9% (0.0%)	None	na	na	na
CTA	100% (221/221)	100% (38/38)	94.7% (71/75)	75% (3/4)	98.6%	97.6%	5 (3)/79	6.3% (3.8%)	None	na	na	na
MER	98.0% (245/250)	97.8% (45/46)	95.0% (38/40)	100% (26/26)	97.6%	98.6%	None	na	None	na	12/374	3.2%
IMP	99.6% (245/246)	100% (16/16)	93.3% (42/45)	100% (19/19)	98.6%	100%	None	na	None	na	9/331	2.7%
ERT	100% (220/220)	na	96.1% (74/77)	na	99.0%	na	3 (2)/77	3.9% (2.6%)	None	na	na	na
AMK	100% (264/264)	97.6% (40/41)	90.9% (30/33)	100% (1/1)	99.0%	97.6%	3 (1)/34	8.8% (2.9%)	1 (0)/305	0.3% (0.0%)	na	na
GEN	99.6% (240/241)	100% (22/22)	98.2% (55/56)	100% (14/14)	99.3%	100%	1 (0)/70	1.4% (0.0%)	1 (1)/263	0.4% (0.4%)	na	na
CIP	100% (176/176)	100% (50/50)	92.5% (99/107)	100% (28/28)	97.2%	100.0%	4 (1)/135	3.0% (0.7%)	None	na	17/375	4.5%
SXT	99.5% (183/184)	94.4% (17/18)	100% (112/112)	100% (17/17)	99.7%	97.1%	None	na	2 (2)/202	1.0% (1.0%)	1/332	0.3%
Organism group	98.2%	96.9%	96.8%	98.4%	97.8%	97.2%						
Overall	98.0%		96.9%		97.7%		31	2.3%	17	0.5%	98	2.1%

^*a*^ n/N, represents the number of results correctly categorized by iFAST divided by the total number of BMD results in the category being assessed; VME, very major error; VME>1, represents the number of VMEs for which the BMD MIC was more than 1 dilution from the breakpoint; aVME, adjusted very major error; ME, major error; ME>1, represents the number of MEs for which the BMD MIC was more than 1 dilution from the breakpoint; aME, adjusted major error; mE, minor error; na, not applicable.

Taken together, the results reported here, in addition to literature evidence, highlight the challenges posed to rapid AST technologies by β-lactam/β-lactamase inhibitor (BL/BLI) combination agents. The overall number of VMEs associated with BL/BLI antibiotics in this work was 18 out of a total of 350 resistant results for such combination agents.

Reproducibility was assessed with 12 isolates: 5 *Escherichia coli*, 5 *Klebsiella pneumoniae*, 1 isolate of *P. aeruginosa*, and 1 isolate of the *E. cloacae* complex. Testing was performed across three sites, in triplicate, on 3 separate days, with different operators at each site. Agreement was 95.7%, above the acceptance criteria detailed in ISO-20776-2:2021. Greater than 95% of the QC results from all sites combined were within the expected range during the evaluation period.

ISO 20776-2:2021 requires analysis to be reported as sensitivity and specificity and as a minimum by organism group (*Enterobacterales* and non-fermenters). iFAST specificity and sensitivity for the *Enterobacterales* was 98.2% and 96.8%, respectively, and 96.9% and 98.4% for the non-fermenters, exceeding the acceptance criteria (≥95% sensitivity and specificity). Overall CA was 97.8% and 97.2% for the *Enterobacterales* and non-fermenters, respectively.

The antimicrobials exhibiting the lowest specificity were aztreonam and ceftazidime. The aztreonam specificity for the *Enterobacterales* was 88.7% (Table 4). According to EUCAST clinical breakpoint tables, version 15 (2025), this antibiotic has a two-dilution susceptible, increased exposure (I) range. When all susceptible and susceptible, increased exposure (I) results were grouped together, the aztreonam specificity increased to 99.0%. The ceftazidime specificity for the non-fermenters (*P. aeruginosa*) was 86.8%. This was due to five mEs that were unresolved by DRT, where all isolates tested susceptible, increased exposure (I) by BMD and resistant by iFAST. Consensus BMD MICs of ceftazidime for these isolates ranged from 0.5 to 8 mg/L. The lowest sensitivity rates were observed for amoxicillin-clavulanic acid (91.8% for the *Enterobacterales*), ceftazidime-avibactam (66.7% for the non-fermenters), ceftolozane-tazobactam (75% for the non-fermenters), amikacin (90.9% for the *Enterobacterales*), and ciprofloxacin (92.5% for the *Enterobacterales*) (Table 4). Several isolates that tested resistant to these agents by BMD and susceptible by iFAST (VMEs) had consensus BMD MICs within ±1 dilution of the breakpoint. When these results were removed from the analysis, sensitivity increased to 96.6%, 100%, 93.8% and 95.2% for amoxicillin-clavulanic acid, ceftazidime-avibactam, amikacin, and ciprofloxacin, respectively. The relatively low sensitivity observed for ceftolozane-tazobactam among non-fermenters was attributable, in part, to the small number of resistant isolates available for this antimicrobial-organism group (*n* = 4).

For the entire panel of antibiotics, the median iFASTone TTR was 3 h 50 min (range, 3 h 18 min–5 h 45 min), and the median disk diffusion TTR was 34 h 10 min (range, 20 h 53 min–76 h 8 min). Compared with disk diffusion, iFASTone reduces the time to result by a median of 30 h 20 min. The time to result is under 4 h, with an iFASTone instrument run time of less than 3 h. The iFASTone system provides one of the fastest phenotypic susceptibility results currently available among rapid AST technologies (9–22). In routine clinical use, it is unlikely that the entire panel of antibiotics would be measured, giving even faster TTR. The iFASTone should provide same shift results even for blood culture samples that flag positive halfway through a working day. The iFASTone system has the potential to transform patient pathways by compressing the therapeutic uncertainty window. By delivering phenotypic AST results in under 4 h directly from PBCs, the system enables same-shift targeted adjustments and proactive antimicrobial stewardship team involvement, particularly during core working hours.

Clinical performance of the iFASTone system was broadly comparable with other rapid AST technologies (9–22). However, the system offers several operational advantages, including fully automated fluidic handling, label-free detection, and elimination of the need for preparation of a 0.5 McFarland suspension. Instead, the system automatically dilutes samples to approximately 5 × 10⁵ CFU/mL, simplifying workflows and reducing hands-on time.

The iFASTone is a self-contained instrument with a low-cost antibiotic plate consumable, unlike some systems such as the FASTinov, which requires a commercial optical flow cytometer for measurements. The iFASTone gram-negative kits can be stored at room temperature and therefore can be shipped without cold-chain logistics, facilitating deployment in settings where cold-storage infrastructure may be limited. Depending on laboratory workflows and the number of antimicrobial agents tested, between 5 and 15 clinical samples can be processed during an 8 h shift. At this throughput, a single instrument would be sufficient to process all gram-negative PBCs in most United Kingdom microbiology laboratories.

The iFASTone gram-negative panel comprises 15 antimicrobial agents spanning the principal therapeutic classes used for the management of BSIs in United Kingdom practice (aminopenicillins and BL/BLI combinations, third-generation cephalosporins, carbapenems, aminoglycosides, fluoroquinolones, and trimethoprim-sulfamethoxazole). The test is performed directly from PBC bottles for *Escherichia coli*, *Klebsiella pneumoniae*, *Klebsiella oxytoca*, *E. cloacae* complex, *Serratia marcescens*, *P. aeruginosa,* and *A. baumannii*, enabling early escalation/de-escalation decisions across the dominant *Enterobacterales* and non-fermenter bloodstream pathogens. These organisms represent some of the most frequent gram-negative causes of BSIs in the United Kingdom and exhibit diverse and evolving resistance mechanisms, making comprehensive susceptibility testing essential. The panel includes both first-line therapies and reserve agents intended for the treatment of infections caused by multidrug-resistant strains. With a 15-agent menu, the iFASTone gram-negative panel provides broader coverage than both the Accelerate PhenoTest BC gram-negative kit and the FASTinov FASTgramneg kit, which both comprise 12 antibiotics. Compared with the more expansive panels offered by VITEK REVEAL and the Q-linea ASTar systems, which include >20 gram-negative antibiotics to approximate a full routine susceptibility profile, the iFASTone system offers a streamlined menu focused on clinically actionable agents for early BSI management.

### Limitations of the study

There are some noteworthy limitations of the present study. Firstly, although conducted across three United Kingdom laboratories, the study sites were located in the South and South West of England and may not have captured the full diversity of pathogen epidemiology, antimicrobial resistance prevalence, and laboratory workflows encountered across the United Kingdom. However, the isolate collection included representatives of the dominant acquired β-lactamase families of clinical importance in *Enterobacterales* in the United Kingdom and globally, namely, CTX-M ESBLs (e.g., CTX-M-15), plasmid-mediated AmpC enzymes (e.g., CMY-6), and the carbapenemase “big 5” (KPC, NDM, VIM, IMP, and OXA-48-like). Secondly, all stock and contemporary isolates were tested using contrived positive blood cultures, with stock isolates included to ensure adequate representation of specific species and susceptibility profiles. While this approach is commonly used in evaluations of AST systems, contrived samples may not fully reproduce the physiological conditions of clinical bloodstream infections and typically lack the biological complexity of clinical specimens. Thirdly, enrichment for resistant phenotypes may have introduced spectrum bias and over-represented uncommon resistance mechanisms relative to routine clinical practice. Finally, certain antimicrobial-organism combinations remained underrepresented despite targeted inclusion strategies, thereby limiting the generalizability of performance estimates for those agents.

### Conclusions

Overall reproducibility was 95.7%, indicating consistent performance across runs and sites. Across all organism groups, the overall sensitivity and specificity were 96.9% and 98.0%, respectively, corresponding to low rates of false-susceptible and false-resistant results. Overall CA was 97.8% and 97.2% for the *Enterobacterales* and non-fermenters, respectively. These values are above commonly recognized thresholds for acceptable clinical performance. Note that CA and associated terminology have been removed from the second edition of ISO 20776-2.

For *Enterobacterales*, sensitivity was 96.8%, with specificity of 98.2%, supporting reliable use of the iFASTone system for this clinically important organism group, including stewardship of critical antimicrobial agents. Performance for non-fermenters was also acceptable, with sensitivity of 98.4% and specificity of 96.9%. The slightly lower specificity is associated with breakpoint-adjacent phenotypes and is consistent with the recognized challenges these organisms present for rapid AST systems.

Future developments will focus on expanding the clinical and technical capabilities of the iFASTone system, including improved algorithms incorporating machine learning. Planned work includes the introduction of quantitative AST for critical first-line and escalation agents, particularly piperacillin-tazobactam and carbapenems. Additional priorities include the clinical evaluation of expansive gram-positive and fungal panels, the development of a specialist *Neisseria gonorrhoeae* panel (30), and rapid testing of urinary tract infections (25). Further research will also explore the potential application of the iFAST technology for phage antimicrobial susceptibility testing.

## Data Availability

The data that support the findings of this study are available at https://doi.org/10.5258/SOTON/D3931.
